# Obeticholic Acid and Other Farnesoid-X-Receptor (FXR) Agonists in the Treatment of Liver Disorders

**DOI:** 10.3390/ph18091424

**Published:** 2025-09-22

**Authors:** Stefano Fiorucci, Ginevra Urbani, Eleonora Distrutti, Michele Biagioli

**Affiliations:** 1 Department of Medicine and Surgery, University of Perugia, 06123 Perugia, Italy; ginevra.urbani@dottorandi.unipg.it (G.U.); michele.biagioli@unipg.it (M.B.); 2 SC di Gastroenterologia ed Epatologia, Azienda Ospedaliera di Perugia, 06123 Perugia, Italy; eleonora.distrutti@ospedale.perugia.it

**Keywords:** obeticholic acid (OCA), primary biliary cholangitis (PBC), ursodeoxycholic acid (UDCA), metabolic dysfunction-associated steatohepatitis (MASH), bile acid receptors (BARs)

## Abstract

The Farnesoid-X-receptor (FXR) is a bile sensor involved in the regulation of bile acid homeostasis, fibrosis, inflammation, and metabolism. Obeticholic acid (OCA), a semisynthetic derivative of chenodeoxycholic acid (CDCA), initially named 6-ethyl-CDCA or INT-747, is the first in a class of FXR ligands that have been approved for clinical use for the treatment of patients with primary biliary cholangitis (PBC) who are unresponsive or intolerant to ursodeoxycholic acid. In this narrative review, we will examine the current status and future perspective of clinical use of OCA. Based on results from phase 2 and 3 clinical trials, OCA received a conditional market approval for its use as a second-line treatment for the management of PBC in 2016. However, concerns over drug (OCA)-induced liver injury (DILI), including hepatic decompensation in cirrhotic and non-cirrhotic PBC patients, have led to discontinuation of OCA commercialization in the EU, but not in North America and the UK, in 2024. Based on positive results from preclinical models, OCA has been investigated also for the treatment of metabolic dysfunction-associated steatohepatitis (MASH). Results from phase 2 and 3 trials, however, have shown that while OCA reduces liver fibrosis, the beneficial effects on steatosis are marginal, thus preventing its clinical approval under the current regulatory guidelines. Here, we review potential applications of OCA in PBC patients in the context of a highly competitive therapeutic landscape, generated by the approval for clinical use of safer and effective second-line therapies, including PPARs agonists such as elafibranor and seladelapar and increased off-label use of fibrates. The current status of development of second-generation FXR agonists such as cilofexor, tropifexor, and vonafexor and their potential in the treatment of liver fibrosis in MASH will be discussed and compared to recently approved therapies, resmetirom, and semaglutide, a GLP-1 agonist. Finally, since some of the novel candidates for treating MASH, have shown limited efficacy on liver fibrosis, we suggest that development of combinatorial therapies based on FXR ligands and agents acting on different molecular targets might offer the opportunity for the repositioning of drug candidates whose development has been abandoned for insufficient efficacy, minimizing/recovering costs linked to drug development.

## 1. Introduction

The farnesoid-x-receptor (FXR-NR1H4) is a transcription factor that was originally identified by Forman and colleagues in 1995 [1] as the putative receptor for farnesol, an intermediate in the mevalonate pathway. As other nuclear receptors, the protein structure FXR consists of an N-terminal DNA-binding domain (DBD), a hinge region, and a C-terminal ligand-binding domain (LBD) [2]. FXR is expressed as four different isoforms from a single locus in both humans and mice [3]. These isoforms differ in their activation function domain 1 (AF-1) at the N-terminus, while the DBD, which presents two zinc finger motifs, allowing for the recognition of specific DNA sequences, is the most conserved region. The DBD of FXR recognizes specific FXR response element (FXR-RE) on target genes, promoting the displacement of co-repressors, such as N-Cor, and recruitment of specific co-activators that regulate the transcription of FXR target genes in a tissue-specific manner [2]. FXR binds to these FXR-REs as an heterodimer with the Retinoid-X-receptor (RXR) [4], while direct binding as a single FXR homodimer is unusual. While FXR-REs have been identified throughout the whole human genome, they are especially clustered in genes that are involved in bile acid metabolism and lipid homeostasis. The FXRα2 isoform binds approximately 89% of FXR binding sites, and the everted repeat (ER)-2 motif represents the most common FXR-RE detected in the human liver [5]. FXR was “de-orphanized” as the first bile acid receptor by three independent groups in 1999 [6,7,8], and chenodeoxycholic acid (CDCA) was identified as the most potent endogenous activator of FXR in humans, with a EC_50_ of 10 µM [6,7,8]. The other major primary bile acids, cholic acid (CA) and secondary bile acids such as lithocholic acid (LCA) and deoxycholic acid (DCA), can also activate FXR, although they are considerably less potent than CDCA [9]. In mice, since CDCA is converted into muricholic acids (MCAs), the most potent FXR agonist is CA. These species-specificities are important when translating results from rodents to humans, since some MCAs are more hydrophilic than CDCA and function as FXR antagonists [10].

The first synthetic ligand of FXR is GW4064, an isoxazole derivative that was originally discovered by Maloney et al. in 2000 [11]. GW4064 is still used as a toll for in vitro studies but was never advanced for clinical purposes. Various isoxazole-based derivatives, however, such as cilofexor, nidufexor, and others, have been developed and have reached the clinical development stage [12,13,14,15]. The discovery of GW4064, along with the generation of FXR knockout (FXR^−/−^) mice by the Gonzalez’s group [16], have been critical steps in decoding FXR signaling in physiology and pathology [2].

**Figure 1:**
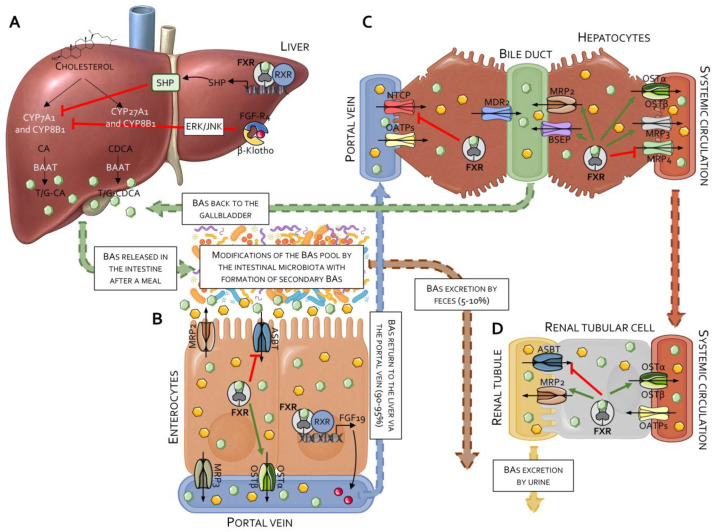
**Effects of FXR in bile acid metabolism.** (**A**) Bile acids are synthesized in the liver from cholesterol through the action of the enzymes CYP7A1 and CYP8B1 (classical pathway), leading to the formation of cholic acid (CA), or alternatively via CYP27A1 and CYP8B1 (alternative pathway), resulting in the production of chenodeoxycholic acid (CDCA). Primary bile acids are then conjugated and stored in the gallbladder to be released into the intestine postprandially. Hepatic activation of FXR leads to CYP7A1 downregulation via induction of the small heterodimer partner (SHP). Moreover, FXR activation in enterocytes promotes the release of FGF19, which binds to the hepatic FGFR4/β-Klotho receptor complex and further inhibits CYP7A1 expression through ERK/JNK signaling pathways. (**B**) In the intestine, bile acids undergo extensive biotransformation by the gut microbiota, resulting in the formation of secondary bile acids. The majority of bile acids entering the intestine (90–95%) are reabsorbed by enterocytes and brought back to the liver via the portal vein. FXR activation in enterocytes inhibits the ASBT, which mediates bile acid uptake from the intestinal lumen, while inducing the expression of the OSTα/β heteromeric transporter that is responsible for basolateral efflux of bile acids into the portal circulation. A minor fraction of bile acids that are not reabsorbed are then excreted in the feces. (**C**) Bile acids that are absorbed by intestinal cells reach the liver through the portal circulation. In the liver microcirculation, bile acids are taken up by hepatocytes, primarily via NTCP and organic anion-transporting polypeptides (OATPs). They are then secreted into the bile canaliculi by apical transporters such as MDR2, MRP2, and BSEP. FXR activation in hepatocytes suppresses NTCP expression while promoting the activity of MRP2 and BSEP. Furthermore, activated FXR upregulates MRP3 and OSTα/OSTβ, which mediate bile acid efflux from basolateral membranes of hepatocytes into the systemic circulation. (**D**) Via the systemic circulation, bile acids reach the kidney, where they are partially excreted in the urine. FXR activation in renal tubular cells inhibits ASBT, thus limiting bile acid reabsorption from the pre-urine, while promoting the expression of MRP2 and OSTα/OSTβ and re-uptake of bile acids into the systemic circulation.

In addition to direct binding to FXR-RE on target genes, FXR exerts some of its regulatory effects by promoting the transcription of various regulatory mediators. The best characterized of these regulatory factors is the small heterodimer partner (SHP, NR0B2), an atypical nuclear receptor that lacks the DNA-binding domain and function as a co-repressor for some FXR-regulated genes (Figure 1) [17], including CYP7A1, cholesterol 7 alpha-hydroxylase, the rate-limiting enzyme for the synthesis of primary bile acids from cholesterol [18]. In liver cells, activation of FXR promotes the transcription of SHP that binds to the Liver Receptor Homolog-1 (LRH-1), a transcriptional factor that positively regulates CYP7A1 [18], creating a liver-centered feedback loop that contributes to regulating the flow of cholesterol toward bile acids [19]. In addition to this mechanism, in 2006, Inagaki et al. [20] reported that activation of intestinal FXR promotes the release of an enterokine, Fibroblast Growth Factor (FGF)-19, which also represses CYP7A1 expression/function in hepatocytes. FGF19 is released by ileal cells in response to activation of intestinal FXR in the late post-prandial phase, and once released (Figure 1), it is transported back to the liver through the portal circulation. In the liver, FGF19 (FGF15 is its mouse ortholog) binds to FGF Receptor 4 (FGF-R4), expressed on the cell membrane of hepatocytes, promoting the assembly of an FGF-R4/βklotho complex that, in its turn, represses CYP7A1 expression and function. Thus, FGF19 is a key enterokine in the FXR-dependent intestinal–liver endocrine network [2]. Thus, while FXR-SHP exerts an immediate feedback regulation of CYP7A1 expression/activity upon changes in intracellular levels of bile acids in hepatocytes, the FXR-FGF19/FGF-R4 axis functions as a regulatory mechanism in the late digestion phase. In aggregate, these findings indicate that FXR functions as a bile acid sensor in the entero-hepatic tissues, and its activation in vivo represses bile acid synthesis. Because inhibition of bile acid synthesis impacts bile flow and cholesterol excretion, it has been suggested that FXR antagonists [21,22,23,24] might also be of clinical utility, pointing toward potential drawbacks of FXR activation in clinical settings.

## 2. FXR Agonists in Clinical Development

**Figure 2:**
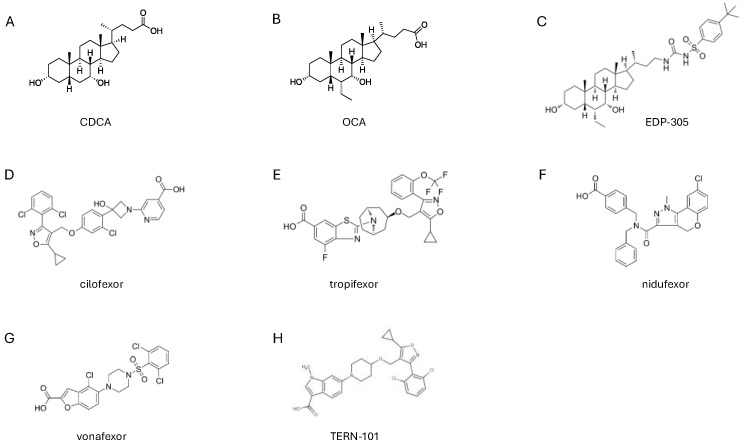
**Structure of FXR agonists that are currently assessed in clinical trials.** Chemical structures of (**A**) CDCA, (**B**) OCA, (**C**) EDP-305, (**D**) cilofexor, (**E**) tropifexor, (**F**) nidufexor, (**G**) vonafexor and (**H**) TERN-101 FXR agonists.

Obeticholic acid (OCA) [25] is the first, and so far the only, FXR agonist that is approved for clinical use. Chemically, OCA, or 6α-ethyl-3α,7α-dihydroxy-5-cholan-24-oic acid, is a semisynthetic derivative of CDCA, 3α,7α-dihydroxy-5-cholan-24-oic acid (Figure 2). The discovery of OCA was due to two different teams of investigators at the University of Perugia, a chemistry team lead by Prof. Roberto Pellicciari and a gastroenterology/hepatology team lead by Prof. Stefano Fiorucci [25]. OCA was originally termed 6-ethyl (E)CDCA. In 2003, the compound was transferred to Intercept Pharmaceuticals (Intercept Pharmaceuticals Ltd. (Morristown, NJ, USA), a Delaware company, which was cofounded by Stefano Fiorucci, Roberto Pellicciari, and Mark Pruzanski in 2003) and christened as INT-747 [26,27,28,29]. OCA activates FXR at an EC_50_ of 300–600 nM in transactivation assays in HepG2 cells that are transfected with human FXR, and as low as ~100 nM in cell-free assays [25]. Since CDCA activates FXR with an EC_50_ of ≈10 μM [30], OCA is ≈20- to 33-fold more potent than its natural counterpart. However, these in vitro potencies might not translate to different pharmacological effects in vivo due to distinct pharmacokinetic properties of CDCA and OCA [31]. In addition to FXR, OCA might also activate GPBAR1 (TGR5) at an EC_50_ of 0.5–8 μM [32]. In accordance with this view, we have shown that OCA releases GLP-1 (a GPBAR1-regulated gene) from L cells in vivo and Glutag cells in vitro [33,34].

OCA exerts a number of regulatory effects on liver cells, which are described in Figure 1 and are fully consistent with the hypothesis that it can activate liver FXR in vitro and in vivo.

In the last two decades, OCA (INT-747) has been extensively investigated for its antifibrotic, metabolic, and immunomodulatory activities in an impressive number of preclinical models [35]. Some of these studies and their main findings are summarized in Table 1.

**Figure 3:**
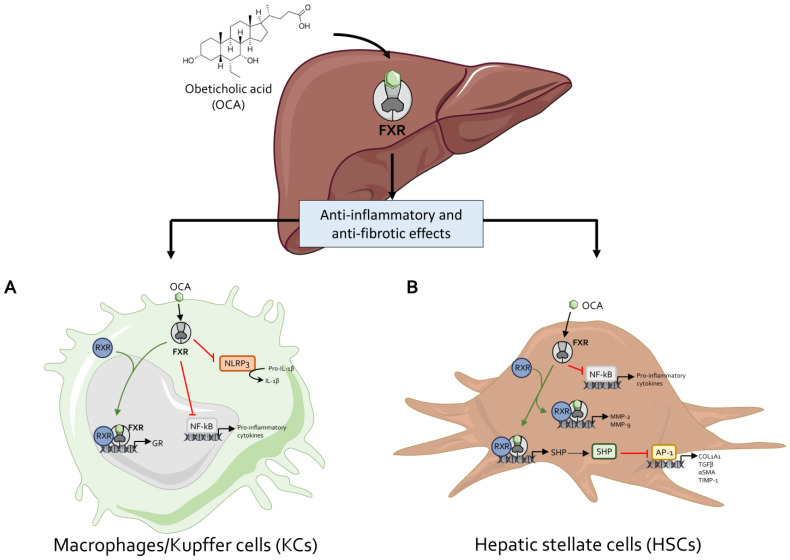
**OCA exerts anti-inflammatory and antifibrotic effects via FXR**. Obeticholic acid (OCA) exerts hepatoprotective effects by modulating both inflammatory and fibrogenic pathways through activation of the farnesoid X receptor (FXR) in hepatic macrophages/Kupffer cells (KCs) and hepatic stellate cells (HSCs). (**A**) In macrophages, FXR activation by OCA suppresses NF-κB signaling, thereby attenuating the transcription of multiple pro-inflammatory cytokines. In addition, OCA reduces NLRP3 inflammasome activation, leading to decreased IL-1β production. FXR activation also promotes transcription of the glucocorticoid receptor through FXR/RXR heterodimer formation. (**B**) In HSCs, FXR ligation by OCA drives heterodimerization with RXR, resulting in upregulation of MMP-2 and MMP-9, which facilitate extracellular matrix degradation. Moreover, FXR/RXR signaling induces SHP expression, which represses AP-1 activity and consequently downregulates profibrotic genes, including COL1A1, TGF-β, α-SMA, and TIMP-1. Finally, FXR activation in HSCs also inhibits NF-κB, thereby reducing the release of pro-inflammatory mediators.

To summarize, while these studies invariably indicate that OCA exerts beneficial effects in a range of animal models of liver metabolic and inflammatory disorders, the core finding is that OCA reduces liver fibrosis and, to some extent, ameliorates liver steatosis. In contrast, the effects exerted by OCA on lipoprotein metabolism were found to be less consistent across the various models, and, while some studies have reported positive effects on circulating levels of LDL, cholesterol, and triglycerides, others have shown no effects on cholesterol and a reduction in HDL, an effect that has been confirmed extensively in clinical studies (see below). Additionally, while OCA reduces intestinal inflammation and modulate innate immunity in models of inflammatory bowel disease (IBD), there is no evidence that these effects occur in clinical settings. In conclusion, while these preclinical studies have been essential for identifying therapeutic areas for OCA and have shown a potential role for FXR agonism in modulating liver fibrosis (this finding has been confirmed in clinical trials), the effect exerted by OCA on lipid metabolism is less consistent (Figure 3). Finally, it should be noted that none of these studies has ever detected the development of itching in OCA-treated animals.

### 2.1. Clinical Development of OCA

In 2006, OCA, with code name INT-747, entered a phase 1 clinical development trial and, following two successful phase 2 and 3 trials, in 2016, OCA (brand name OCALIVA), at a daily dose of 5–10 mg, was granted conditional approval by the Food and Drug Administration (FDA) and the European Medicines Agency (EMA) as a second-line treatment of patients with primary biliary cholangitis (PBC) [67,68] (Table 2) who had an inadequate response to UDCA or were unable to tolerate it [67,68]. Its approval was based on the beneficial effect exerted by OCA on a surrogate biomarker, i.e., levels of alkaline phosphatase (ALP), as the result of an accelerated approval track. In the meantime, OCA was investigated for its potential role in treating steatosis and fibrosis in patients with non-alcoholic steatohepatitis (NASH), a condition that in 2023 was renamed as metabolic dysfunction-associated steatosis (MASLD) or steatohepatitis (MASH) [69]. The following part of this review presents the results of clinical studies on OCA in PBC and MASH.

### 2.2. OCA in the Treatment of Primary Biliary Cholangitis (PBC)

PBC is a progressive, immune-mediated, cholestatic liver disease characterized by chronic symptoms such as fatigue, pruritus, and sicca syndrome. If left untreated, PBC can progress to biliary cirrhosis and hepatocellular carcinoma. The introduction of ursodeoxycholic acid (UDCA) in the 1980s represented a major therapeutic breakthrough in PBC by achieving symptom control, particularly of itching, and slowing disease progression while improving transplant-free survival, particularly when therapy is started in early-stage PBC [70,71]. However, some PBC patients fail to reach a complete biochemical response or are intolerant to UDCA. The proportion of patients that fail to reach a complete biochemical response to UDCA varies significantly according to the criteria used to define “biochemical response” and ranges from 5% to 40% in controlled clinical trials, but it is generally lower in real-world settings. A relatively recent study using different scoring systems reported that an incidence of incomplete biochemical response to UDCA occurred in 30.7% of patients using the Barcelona liver clinic criteria, in 35.3% of PBC patients using the Paris I criteria, in 53.7% of patients using the Paris II criteria, and in 36.4% using the now widely accepted Globe score [70,72,73,74,75]. Adopting the Paris II criteria and adjusting for age and sex, the risk of a biochemical incomplete response (BIR) was 25% higher for patients with cirrhosis at diagnosis, 35% higher for patients with elevated gamma-glutamyl transferase (γGT), and 5% higher for those with elevated and alkaline phosphatase (ALP) [76].

Since UDCA non-responders have a poorer prognosis compared to those who respond well to UDCA and may experience faster disease progression and carry an increased risk of developing complications, several second-line therapies have been proposed for the treatment of UDCA non-responders [77].

**Figure 4:**
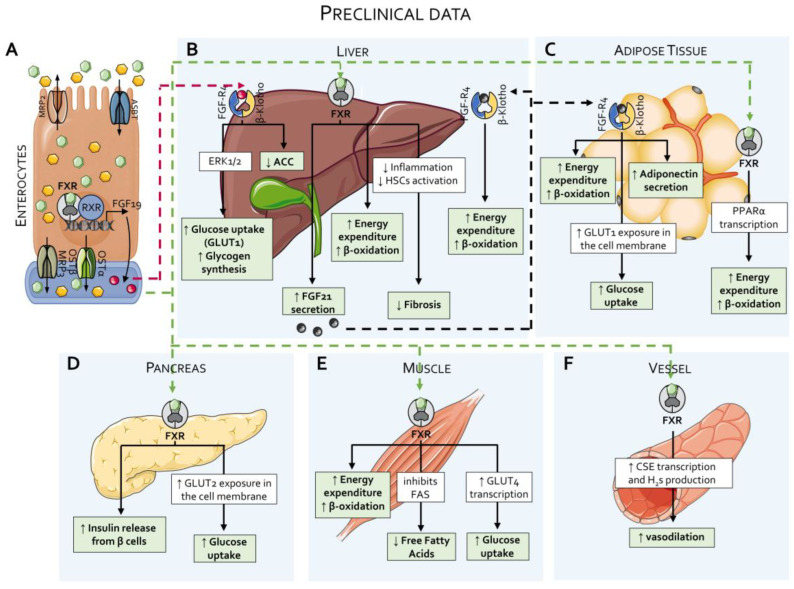
**Effects of FXR agonists on lipid and glucose metabolism: preclinical data.** (**A**) As detailed in Figure 2, approximately 90–95% of bile acids are reabsorbed by enterocytes from the intestinal lumen and returned to the portal circulation via specific transporters. In enterocytes, activation of FXR by primary bile acids induces the production of Fibroblast Growth Factor (FGF)-19 and enhances the activity of the OSTα/OSTβ transporter complex. (**B**) Through the portal circulation, bile acids and FGF19 first reach the liver. In the liver, FXR activation enhances energy expenditure and β-oxidation, suppresses inflammation and hepatic stellate cell (HSC) activation—thereby reducing liver fibrosis—and stimulates the production of FGF21. Hepatocytes also express the FGFR4/β-Klotho receptor complex, which binds FGF19 and promotes glucose uptake and glycogen synthesis via the ERK1/2 signaling pathway, while concurrently inhibiting acetyl-CoA carboxylase (ACC) activity. The same receptor complex binds FGF21, further enhancing energy expenditure and β-oxidation. (**C**) In white adipose tissues, FXR activation induces the transcription of PPARα, thereby increasing energy expenditure and β-oxidation. Adipose tissue also expresses the FGFR4/β-Klotho complex, which, upon binding to FGF21, elicits multiple metabolic effects, including increased energy expenditure and β-oxidation, enhanced glucose uptake via the glucose transporter protein type (GLUT) 1, and elevated adiponectin secretion. (**D**) In the pancreas, FXR activation promotes insulin secretion from β-cells and increases glucose uptake through the GLUT2 transporter. (**E**) In skeletal muscle, FXR activation enhances energy expenditure, β-oxidation, and glucose uptake via GLUT4, while simultaneously suppressing de novo synthesis of free fatty acids. (**F**) FXR also exerts vasodilatory effects in the vascular system by inducing hydrogen sulfide (H_2_S) production through the action of cystathionine γ-lyase (CSE).

As described above, FXR functions as a bile acid sensor that represses synthesis of primary bile acids by inhibiting the expression function of CYP7A1, an enzyme that catalyzes the first and rate-limiting step in the cholesterol-to-bile acid synthesis pathway in the liver, while promoting the excretion of bile acids from hepatocytes by activating feed-forward secretion mechanisms (Figure 1). Inhibition of CYP7A1 by FXR agonism is mediated by two independent mechanisms. In vitro and in vivo studies have confirmed that OCA regulates both mechanisms. Exposure to OCA increases SHP, which binds to LRH1 acting as a negative regulator CYP7A1, thus reducing bile acid synthesis [18,41,78]. In addition, in vivo administration of OCA promotes the release of FGF19 [68] from the intestine, which represses CYP7A1 after binding to the FGFR4/β-Klotho complex on hepatocytes [79]. In addition to its repressing effects on bile acid synthesis, OCA inhibits the expression of the sodium/bile acid cotransporter (Na^+^-taurocholate co-transporting polypeptide, NTCP), thus preventing bile acid uptake from hepatocytes [25,80], and promotes the expression of feed-forward transporters in hepatocytes, including the bile salt export pump (BSEP) and the multidrug resistance protein 2 (MRP2, also known as MDR2) [25,80], thus increasing the excretion of bile acids from the hepatocytes and promoting the bile flow [41]. OCA also increases an FXR-dependent expression of the organic solute transporter (OST)α/β, thus increasing the excretion of bile acids from the basolateral membrane of hepatocytes [25,80,81]. On the other hand, similarly to endogenous FXR ligands such as CDCA, OCA inhibits the expression of the efflux proteins such as Multidrug Resistance Protein 4 (MRP4/ABCC4), thus reducing the protective effects of this efflux system against dangerous concentrations of bile acids in hepatocytes [25,80,82]. The negative regulation of this basolateral transporter explains why FXR gene ablation or FXR antagonism [23] in mice protects from cholestasis [21] and might explain some of the toxicity of OCA in rodent models of cholestasis [83] (Figure 4). Preclinical studies have shown that OCA attenuates non-obstructive cholestasis. In 2005, we reported that 6-ECDCA (OCA) effectively restores the bile flow in rats who were rendered cholestatic by administration of estrogens [41]. However, this beneficial effect was not maintained in models of obstructive cholestasis [26], such as bile duct-ligated rats. In this later model, exposure to OCA worsened the severity of liver injury and increased mortality [83] (Figure 4).

Based on these preclinical findings, OCA was tested for efficacy in PBC patients (Table 2). Under an FDA-pre-authorized development program, FXR was trialed in PBC patients who were non-responders to UDCA for its efficacy in reducing alkaline phosphatase (ALP), a surrogate marker of disease progression [68], and although its use resulted in various side effects, the most common of which was pruritus (Table 2), OCA was granted clinical approval in the treatment of adult PBC patients who were resistant or intolerant to UDCA in 2016. Pruritus associated with the use of OCA is usually dose-dependent and often requires co-treatments, including co-administration of bezafibrate or rifampicin, dose adjustments, or temporary treatment interruption, with symptoms frequently resolving over time. Permanent discontinuation due to pruritus, however, occurs in 15–35% of PBC patients in various studies. A summary of clinical studies carried out in PBC patients is reported in Table 2.

**Table 2:** Phase 2 and 3 and post-approval trials of OCA in patients with PBC.

Study Group (n. Patients)	UDCA Dose	ALP (UI/L)	ALP Decrease	Pruritus Incidence % (% of Patients with Severe Pruritus)	Ref.
**Phase 2 trial**					[67]
Placebo (38)	15.9	275.2 ± 102.7	3%	50%	
OCA 10 mg (38)	15.9	294.4 ± 149.4	24%	47% (16%)	
OCA 25 mg (48)	15.6	290.0 ± 123.6	25%	85% (24%)	
OCA 50 mg (41)	16.3	286.9 ± 106.2	21%	80 (37%)	
**Phase 3 trial (POISE study)**					[68]
Placebo (73)	15 ± 4	327.2 ± 115	29%	38%	
OCA 5–10 mg (70)	17 ± 5	326 ± 116	77%	56%	
OCA 10 mg (73)	16 ± 5	316 ± 104	77%	58%	
**OCA as monotherapy up to 6 years follow-up**					[84]
Placebo (23)	No		0.8%	35%	
OCA 10 mg (20)	-		53.9%	70% (15% discontinuation)	
OCA 50 mg (16)	-		37.2%	94% (38% discontinuation)	
3-year POISE open-label extension (POISE-OLE) (2019) *					[85]
OCA 10 mg		ALP at 48 months: −95.6 ± 121.1 U/L		77%	
COBALT study ** 2025—Confirmatory study					[86]
OCA 5–10 mg (168)		Primary endpoints met by: 28.6%		78.6%	
Placebo (166)		Primary endpoints met by: 28.6%		51.2%	

* In the POISE-OLE, 193 of 217 patients form the original POISE [33] and were treated for 3 years during the open-label extension. ALP concentrations were significantly reduced when compared with baseline (mean change −105.2 U/L [SD 87.6]), 24 months (−101.0 U/L [SD 98.5]), 36 months (−108.6 U/L [SD 95.7]), and 48 months (−95.6 U/L [SD 121.1]; *p* < 0.0001 for all yearly time points). ** The COBALT study was a confirmatory study. The primary composite endpoint was time to the onset of one of the following events: death, liver transplant, model for end-stage liver disease (MELD) score ≥ 15, uncontrolled ascites, or hospitalization for hepatic decompensation. The authors reported that both functional unblinding and crossover to commercial therapy occurred, particularly in the placebo arm. The study failed to confirm the superiority of OCA over placebo and the primary endpoint.

The beneficial effects of OCA in PBC patients identified in clinical trials have been corroborated by various real-world cohort studies [87]. In general, these studies have confirmed the clinical benefits of OCA, although several limitations on efficacy have been shown. In a relatively large open-label study involving 191 patient from the PBC Italian registry, the response rate was 42.9% according to the Poise criteria but only 11% by the normal range criteria [88]. A lower response was detected in patients with cirrhosis compared to patients without cirrhosis (29.5% vs. 49.2%; *p* = 0.01) as a result of a higher rate of OCA discontinuation (30% vs. 12%; *p* = 0.004), although both groups showed similar ALP reductions (29.4% vs. 34%; *p* = 0.53). Thirty-three patients (17%) prematurely interrupted OCA because of adverse events, and eleven of them experienced serious adverse events. In 67% of patients, the major cause for OCA discontinuation was treatment-induced pruritus.

An indirect approach to test the efficacy of OCA in PBC patients was adopted in 2022 by using a propensity-score-matched analysis comparing data from the POISE and POISE-OLE clinical trials with two international PBC registries [89]. The study aimed to evaluate the time to first occurrence of liver transplantation or death in PBC patients who were treated with OCA in the POISE trial and in its open-label extension (POISE-OLE) in comparison to non-OCA-treated PBC patients retrieved from the Global PBC and UK-PBC registries. The comparison showed that OCA treatment substantially reduced the likelihood of liver transplantation and death in patients with PBC (POISE vs. Global PBC, hazard ratio [HR] = 0.29, 95% confidence interval [CI] = 0.10–0.83; POISE vs. UK-PBC, HR = 0.30, 95% CI = 0.12–0.75) [68]. As mentioned in Table 2, however, these data were not matched by results from the COBALT study, a confirmatory study that was designed to comply with FDA and EMA requirements, confirming the difficulty of comparing clinical trials with results from real-word practice.

In aggregate, these studies suggest that a proportion of PBC patients who are treated with OCA 5–10 mg and 10 mg, ranging from 24% to 77%, achieve a biochemical reduction in ALP, although a robust percentage of patients develop pruritus, which is the main cause for treatment discontinuation. A recent metanalysis pointed out that the relative risk (RR) for developing new onset pruritus ranges from 1.43 to 1.79 in PBC patients treated with OCA 5–10 mg or 10 mg, in comparison with 0.30 of PBC patients treated with seladelpar and 0.77 in patients treated with elafibranor [77], which were approved as second-line therapies for PBC in 2024.

### 2.3. OCA as Monotherapy in PBC

A study comparing OCA as a monotherapy in PBC patients was reported in 2018 [84]. This was small phase 2 trial in which PBC patients firstly were randomized to receive placebo or OCA 10 mg/die, 50 mg/die as monotherapy for 3 months and then, after the termination of randomized trial, followed for 6 years in an open-label extension of the trial. During this period, patients did not receive UDCA. The results of the study indicated that while OCA monotherapy significantly improved ALP and other biochemical markers that are predictive of improved long-term clinical outcomes in the double-blind part of the study and maintained the beneficial effects over time in the open-labeled phase of the trial, the incidence of pruritus was very high, occurring in 35% of PBC patients who were administered placebo but 70% and 94% of PBC patients who were administered 10 and 50 mg/day OCA, respectively [84]. Therapy discontinuation caused by side effect onset occurred in 15% of patients and 38% of patients receiving OCA 10 and 50 mg, respectively [88]. The median time for onset of pruritus was 33, 14, and 6 days in the placebo, OCA 10 mg, and OCA 50 mg groups. In summary, the use of OCA as a monotherapy associates with severe pruritus and a very high rate of therapy discontinuation.

#### 2.3.1. Why Does OCA Cause Pruritus?

Several mechanisms have been suggested to explain why OCA causes itching. While none of the current hypotheses has been confirmed in dedicated clinical trials, the activation of Mas-related G-protein-coupled receptor member X4 (MRGPRX4), a G protein-coupled receptor (GPCR) for bile acids that is expressed on a subset of itch-sensing neurons, is gaining growing attention [90,91,92]. MRGPRX4 is activated by bile acids, and MRGPRX4 antagonists are currently proposed for treating pruritus in cholestatic diseases [90,91,92]. In vitro studies have shown that OCA binds and activates MRGPRX4 [92]. EP547, a selective MRGPRX4 inhibitor, is currently undergoing a phase 2 trial, designed to evaluate its safety, efficacy, and tolerability in subjects with PBC- or PSC-derived cholestatic pruritus [93]. An alternative explanation is that OCA might promote the release of IL-31, an FXR- regulated pruritogenic cytokine [94]. Serum IL-31 levels are also increased by cilofexor in NASH, PSC, and PBC patients who are treated with this FXR agonist, suggesting a potential explanation for itching caused by FXR ligands [94]. Additionally, it has been suggested [95] that OCA might activate GPBAR1, a pruritogenic receptor in mice [96]. However, this hypothesis has been abandoned, since GPBAR1 does not mediate the itching response to bile acids in humans [90].

#### 2.3.2. Biomarkers of OCA Activity In Vivo: FGF19 and 7α-Hydroxy-4-Cholesten-3-One (C4)

FGF19, a member of the FGF family, is released from ileal enterocytes in response to intestinal FXR activation [20]. Once released, FGF19 enters the portal circulation, reaching the liver where it binds to and activates the FGFR4/β-Klotho complex on hepatocytes mebranes [97,98]. This activates a series of MAP-kinases (e.g., ERK-1 and ERK-2) and suppresses CYP7A1 expression. Like FXR, FGF19 has strong metabolic effects, as it suppresses the insulin-stimulated expression of lipogenic enzymes such as Sterol Regulatory Element Binding Protein (SREBP)-1c and fatty acid synthase (FAS) in the liver, stimulates glycogen synthesis, and reduces the expression of gluconeogenic enzymes [98]. Significantly increased levels of FGF19 are detected in PBC patients treated with OCA, even at the dose of 5 mg/die [97,99,100], and therefore FGF19 levels are used as biomarker of OCA activity in clinical trials.

An additional biomarker of FXR activation in the liver is the synthesis of primary bile acids. As described in Figure 1, CA and CDCA are generated in the liver by a chain of enzymes that can be grouped into two main metabolic pathways: the acidic pathway and the alternative pathway [19]. The 7α-hydroxy-4-cholesten-3-one (C4) is an intermediate of bile acid synthesis from cholesterol in the acidic pathway, and its levels in the blood reflect the rate of bile acid synthesis by CYP7A1 in the liver (Figure 1). Elevated C4 levels are a sign of increased bile acid production or bile acid malabsorption, while a reduction in C4 levels in the blood can be used as a biomarker of FXR activation [101,102].

### 2.4. Post-Marketing Surveillance in PBC Patients

In 2017, after less than 2 years of marketing, a cluster of severe side effects, including hepatic decompensation requiring liver transplantation, were linked to the use of OCA in cirrhotic PBC patients. A search of the FDA adverse Events Reporting System (FAERS) in May 2025 retrieved as many as 6488 side effects that are potentially linked to the use of OCA. As many as 2840 of these side effects were judged as severe. The database also shows an excess of mortality in cirrhotic patients taking OCA (https://fis.fda.gov/sense/app/95239e26-e0be-42d9-a960-9a5f7f1c25ee/sheet/45beeb74-30ab-46be-8267-5756582633b4/state/analysis, Accessed on 15 September 2025).

The severity of these effects led the FDA to emit a drug safety communication on 1 February 2018 [*source: FDA–Drug safety communication 2 January 2019*], followed by another warning on 26 May 2021. In these drug safety communications, the FDA communicated a restriction on the use of OCA in “*patients having primary biliary cholangitis (PBC) with advanced cirrhosis of the liver because it can cause serious harm. Some PBC patients with cirrhosis who took Ocaliva, especially those with evidence of advanced cirrhosis, developed liver failure, sometimes requiring liver transplant. Based on the original clinical trials, the FDA believes the benefits of Ocaliva outweigh the risks for PBC patients who do not have advanced cirrhosis. We will continue to monitor and evaluate the clinical benefits and adverse events of Ocaliva*”. In December 2024, a further FDA Drug Safety Communication was released, stating that “*Based on its review of post-market clinical trial data, the U.S. Food and Drug Administration (FDA) identified cases of serious liver injury among patients being treated for PBC with OCA who did not have cirrhosis of the liver. We previously identified that PBC patients with advanced cirrhosis were at risk of serious liver injury when taking Ocaliva and updated the prescribing information to restrict its use in these patients*”. The FDA’s final report demonstrated that some cases of liver injury occurred in patients without cirrhosis and resulted in liver transplants (https://www.fda.gov/drugs/drug-safety-and-availability/serious-liver-injury-being-observed-patients-without-cirrhosis-taking-ocaliva-obeticholic-acid-treat, Accessed on 15 September 2025). Similar warnings were released over the years by the EMA.

### 2.5. The Results of the Confirmatory Study, the COBALT Study: OCA Withdrawal in the EU

On June 2024, the EMA recommended OCA’s marketing authorization to be revoked, because its benefits were no longer considered to outweigh its risks. The drug withdrawal was mostly based on negative results from the “COBALT trial study 747-302”, a randomized clinical trial designed to confirm OCA’s clinical benefits and safety in PBC patients who were resistant or intolerant to UDCA. The composite primary endpoint for intention to treat in the analysis in the COBALT trial was as follows: “time to first occurrence of any of the following events: death (all-cause); liver transplant; model for end-stage liver disease (MELD) score ≥ 15; hospitalization ≥ 24 h for new onset or recurrence of variceal bleed, hepatic encephalopathy (West Haven score ≥ 2), or spontaneous bacterial peritonitis (confirmed by diagnostic paracentesis); or uncontrolled ascites requiring therapeutic paracentesis ≥ 2 times in a month. All events were adjudicated by a blinded committee of experts with adjudication experience who were not involved in the study as investigators, data monitoring committee members, or consultants”. The COBALT trial was terminated in December 2021, because it did not seem feasible to continue the study as designed owing to the impossibility of conducting a placebo-controlled randomized trial of long-term outcomes in a setting of commercially available therapies. At that time, 631 patients had been screened, with 334 patients being randomized (placebo, *n* = 166; OCA, *n* = 168; 78% of recruitment target). In the ITT analysis, there were 48 events each in the OCA (28.6%) and placebo (28.9%) arms (HR, 1.01; 95% CI, 0.68–1.51). Of the 48 patients in the OCA arm who had an event, 31 (64.6%) interrupted OCA treatment for a median of 9 months (interquartile range [IQR], 2–17 months) before the event, and 1 initiated commercial OCA treatment. Among the 48 patients in the placebo arm who had an event, 29 (60.4%) discontinued placebo at a median of 4 months (IQR, 1–10 months) before the event. Thus, the study failed to show that OCA was more effective than placebo in reducing the number of patients whose disease worsened or of those who died, as well as in the overall population in an early-stage PBC group. Moreover, the committee did not consider real-world data or data from supportive studies to be sufficient to confirm the benefits of Ocaliva and were thus not able to counterbalance the negative results of the study 747-302. Accordingly, marketing authorization of OCA was revoked in the European Union (https://www.ema.europa.eu/en/news/ema-recommends-revoking-conditional-marketing-authorisation-ocaliva, Accessed on 15 September 2025).

A recent re-analysis of the COBALT trial has been released in 2025 [85]. In this article, results from the COBALT study were compared to an external database that included 1051 non-OCA patients, identified in the Komodo Healthcare Map database. Using this external control, the authors concluded that while functional unblinding and treatment crossover, particularly in the placebo arm, confounded the intent-to-treat estimate of outcomes associated with OCA in the RCT, comparison with the real-world external trial showed that OCA treatment significantly reduced the risk of negative clinical outcomes.

### 2.6. Other FXR Ligands Currently Investigated in PBC

Various FXR ligands in addition to OCA have been advanced to the clinical stage (Figure 2). These include various isoxazole derivatives, such as ED-305, tropifexor, cilofexor and nidufexor [12], and non-bile acid steroids such as BAR-502 [103]. Tropifexor has been investigated in a phase 2 trial in PBC patients with an inadequate response to UDCA and found to be safe and well tolerated [104]. In this trial, 61 PBC patients were randomized to receive placebo or 30, 60, 90, or 150 μg tropifexor. The results demonstrated that while markers of bile duct injury were reduced with tropifexor after 4 weeks of therapy, pruritus of a mild to moderate severity was observed at the highest tropifexor dose [104,105]. Cilofexor has been investigated in phase 2 and 3 trials in patients with primary sclerosing cholangitis (PSC) in a phase 2 trial. The results of an open-label extension of this phase 2 trial are also available [15]. In the OLE phase, noncirrhotic subjects with large-duct PSC who have completed the 12-week, blinded phase of a phase II study were included in a 96-week OLE with cilofexor 100 mg daily. Safety; liver biochemistry; and serum markers of fibrosis, cellular injury, and pharmacodynamic effects of cilofexor on FGF19, C4, and bile acids were evaluated. The results confirmed positive effects on biochemistry (ALP and γGT) and reduction in the serum levels of C4, along with a small but significant increase in FGF19 plasma levels. However, of the 47 subjects enrolled, 15 (32%) discontinued treatment prematurely due to pruritus [*n* = 5], other adverse events [*n* = 5], and subject decision/investigator discretion [*n* = 5]). These results suggest that the development of FXR agonists in cholestasis needs careful reconsideration.

### 2.7. OCA as a Second-Line Therapy for PBC: The Current Landscape

As mentioned above, despite its withdrawal in the EU, OCA remains a second-line therapy in North America, the UK, and various other countries (see below), although its use is contraindicated in PBC patients with cirrhosis and portal hypertension and considering that severe liver effects might occur also in non-cirrhotic PBC. In addition to these limitations and the risk of developing pruritus, it should be considered that other agents were approved as second-line therapies in PBC patients in 2024 [93], including two peroxisome proliferator-activated receptor (PPAR) ligands: elafibranor [106] and seladelpar [107] (Table 3). These agents were shown to be effective in reducing ALP in UDCA-resistant PBC patients in two large phase 3 trials and have received a conditional marketing authorization for the treatment of PBC in combination with UDCA in adults who have an inadequate response to UDCA alone, or as a monotherapy in those who are unable to tolerate UDCA by FDA in 2024 and by EMA on 20 February 2025.

Seladelpar is a selective PPARδ agonist that has been proven effective as a second-line therapy in PBC at the dose of both 5 mg and 10 mg. In a phase 3 trial (the RESPONSE) using the same criteria for patient inclusion as the POISE study, seladelpar 10 mg daily reached a biochemical response in 61.7% of patients, and 25% achieved normalization of ALP. Aside from the beneficial anticholestatic effects, this study also achieved its prespecified secondary endpoint of pruritus improvement [107]. The beneficial effects of seladelpar were maintained for up to two years, as shown by the open-label extension of the RESPONSE study [108]. These findings have led to conditional approval by the FDA and EMA for seladelpar as a second-line therapy for PBC patients with an inadequate response or intolerance to UDCA. A study on PBC patients with decompensated cirrhosis is ongoing (NCT06051617).

Elafibranor is a PPAR α/δ agonist. In a phase 3 trial (ELATIVE), elafibranor at 80 mg daily achieved a biochemical response rate of 51% over 52 weeks, with 15% of patients achieving ALP normalization. Pruritus improvement as a prespecified secondary endpoint was not achieved, while further analysis using the 5D-Itch or PBC-40 itch domain suggested meaningful changes. These data led to conditional approval of elafibranor by the FDA and EMA as a second-line option for PBC patients with an inadequate response or intolerance to UDCA [106]. Comparing results from three randomized, placebo-controlled trials with 570 participants who were treated with OCA, elafibranor, and seladelpar, a recent network metanalysis [77] found that treatment with elafibranor was associated with higher efficacy in inducing a biochemical response at 52 weeks, while seladelpar was associated with a decreased risk of new-onset pruritus. Obeticholic acid showed similar efficacy to elafibranor and seladelpar, although it was associated with an increased risk of severe adverse events [77].

A possible alternative therapy to elafibranor and seladelpar could be a combination of OCA (in those countries where it is still available) with fibrates. Fibrates have been used extensively in PBC patients in open-label studies. The results from the BEZURSO trial demonstrated that among patients with an incomplete response to UDCA, adding bezafibrate at 400 mg daily resulted in a complete biochemical response in 31% of patients, while another third exhibited ALP normalization after two years. Additionally, liver stiffness decreased, and pruritus tended to improve [111].

The use of fibrates in cholestatic liver diseases is generally safe and well tolerated. However, comprehensive safety data for fibrates are lacking due to their off-label use. In the BEZURSO trial, hepatotoxicity was reported in approximately 6% of patients, some of whom required steroid therapy [111]. Phase 2 trials have shown beneficial effects of combining fibrates with OCA, but this strategy cannot be implemented in countries, including those in the EU, where OCA is not currently available [116]. In any case, the biochemical response achieved with the combination of OCA and fibrates did not show superiority compared with OCA or fibrate alone [116].

## 3. OCA in MASLD/MASH

**Figure 5:**
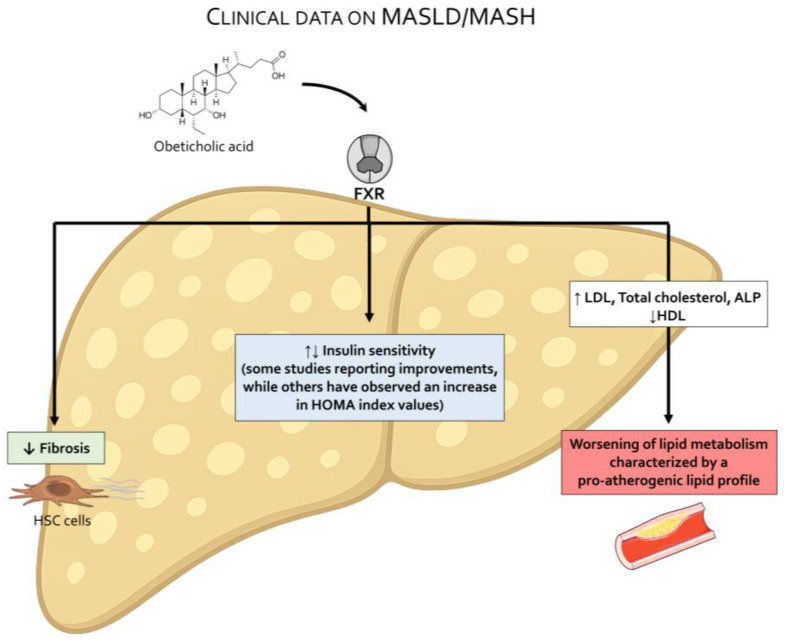
**Treatment with obeticholic acid (OCA) promotes the development of a pro-atherogenic lipid profile in MASH patients.** Results from the FLINT study and the REGENERATE study indicate that OCA improves hepatic fibrosis, while the impact on insulin sensitivity remains unproven. Both the FLINT and REGENERATE study report an increase in insulin levels and HOMA index in OCA-treated patients. OCA treatment worsens lipid metabolism and increases LDL, total cholesterol, and ALP levels, along with a reduction in HDL, thereby promoting a pro-atherogenic lipid profile.

Since its discovery, OCA has been investigated for itspotential role in the regulation of lipid and glucose metabolism, as well as for its efficacy in reversing steatosis and fibrosis in rodent models of chronic liver injury (Figure 4) [26,117,118,119,120], (Figure 5).

Results from two phase 2 trials published in 2013 [101] and 2015 [112] and one phase 3 trial, the REGENERATE trial published in 2019 [113], are available. In 2013, the results from an exploratory study carried out in diabetic patients with presumed NAFLD were published. Investigators randomized 64 patients to receive 25 mg of OCA, 50 mg of OCA, or placebo daily for 6 weeks. Insulin sensitivity (primary outcome) was measured at baseline and after 6 weeks of therapy using a two-stage hyperinsulinemic–euglycemic insulin clamp. Additionlly markers of liver injury and fibrosis assessed by noninvasive liver fibrosis score (Enhanced Liver Fibrosis, ELF) were also measured. The results demonstrated that OCA was well tolerated and ameliorated insulin sensitivity as measured by Homeostatic Model Assessment (HOMA) by 28.0% in the 25 mg group (*p* = 0.019) and by 20.1% in the 50 mg group (*p* = 0.06). However, it caused a statistically significant deterioration of lipid metabolism and worsened the pro-atherogenic lipid profile (Table 4) [101]. Nevertheless, the results were thought to be encouraging, and OCA was advanced to phase 2 and 3 trials to evaluate its efficacy in reversing steatosis and fibrosis in biopsy-proven MASH.

**Figure 6:**
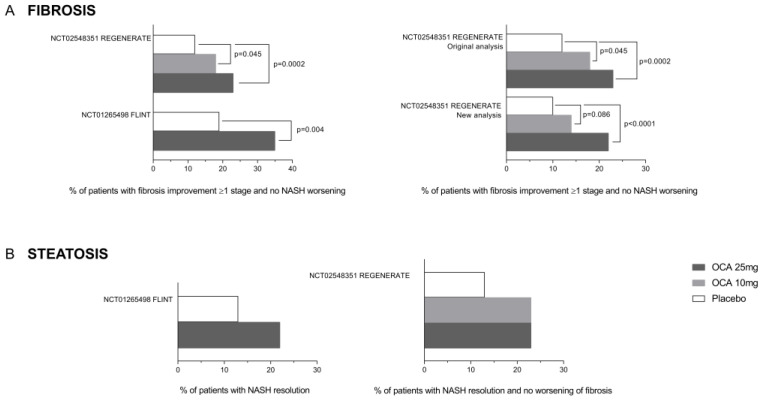
**Effect of obeticholic acid on liver fibrosis and steatosis from the REGENERATE and FLINT clinical trials.** (**A**) The percentage of patients showing fibrosis improvement without NASH worsening increased in a dose-dependent manner in both clinical trials and was confirmed again in the REGENERATE re-analysis. (**B**) No significant differences in terms of NASH resolution (steatosis reduction) were highlighted among different OCA dosages among the clinical trials.

The efficacy of OCA on liver histopathology in patients with biopsy-proven MASH has been investigated in two clinical trials (Table 5, Figure 6).

**Figure 7:**
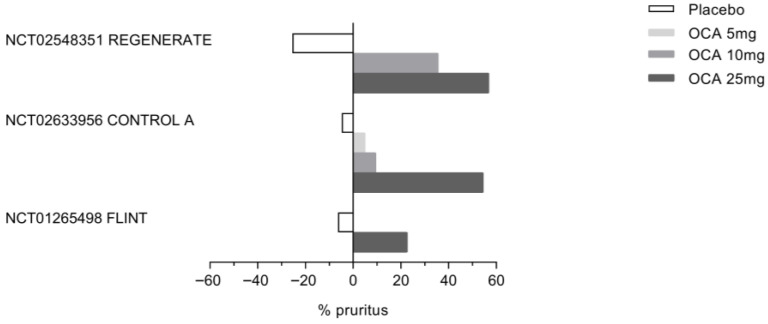
**Incience of Obeticholic acid (OCA)-associated pruritus in three clinical trials: REGENERATE, CONTROL A, and FLINT.** As shown in the figure, the percentage of patients experiencing pruritus in response to OCA increases in a dose-dependent manner in all three clinical trials.

The FLINT study was a randomized, placebo-controlled clinical trial in which 283 patients with histologically proven NASH were randomly assigned to a treatment with placebo (136) or OCA 25 mg/die (135) for 72 weeks. From those patients who concluded the study and were eligible for analysis, 50 (45%) of 110 patients in the OCA group exhibited improvement in liver histology compared with 23 (21%) of 109 patients in the placebo group (biopsies at 72 weeks compared to baseline, relative risk 2.2, 95% CI 1.4 to 3.3; *p* = 0.0002). Patients who had fibrosis improvement at least one stage were 36 (35%) in the OCA group versus 19 (19%) in the placebo group (*p* = 0.004). However, the resolution of NASH occurred in 22 patients (22%) in the OCA group and 13 (13%) in the placebo group, which was close to, but did not reach, a statistically significant value. While OCA reduced AST plasma levels, it increased the Alk. Phos. value by ~15% (*p* < 0.0001). Treating patients with OCA deteriorated their pro-atherogenic lipid profile, as shown by increased levels of total cholesterol and LDL-c and reduced levels of HDL. No changes were observed in glucose plasma levels, while insulin plasma levels and HOMA increased significantly. Among the adverse events, pruritus was the most common, with 33 (23%) of 141 patients in the OCA group developing pruritus compared with 9 (6%) of 142 patients belonging to the placebo group. Moreover, within the OCA group, 24 out of 44 patients with pruritus experienced severe or widespread itching (Figure 7).

On December 2019, the interim results of the phase 3 REGENERATE study were made available [113]. The REGENERATE study was a randomized, double-blind placebo-controlled multicenter trial aimed at evaluating the safety and efficacy of OCA in 931 subjects with stage 2 or 3 liver fibrosis due to NASH. The participants were randomized to receive a placebo, OCA 10 mg/die, or OCA or 25 mg/die once daily for 18 months. Liver biopsies were performed at the end of the study. The primary endpoints for the 18-month interim analysis were fibrosis improvement (≥1 stage) with no worsening of NASH or NASH resolution with no worsening of fibrosis. The study could be considered successful with the meeting of either primary endpoint. First, analyses were carried out by intention to treat (ITT) in patients with F2-F3 fibrosis stages who had received at least one dose of treatment and had reached the 18-month visit. One-stage improvement of liver fibrosis with no worsening of steatosis occurred in 11.9% in the placebo group and in 17.6% and 23.1% of patients who were treated with OCA 10 or 25 mg/die. A statistically significant difference between the placebo and OCA 25 mg/die groups was flagged. Conversely, NASH resolution (based on no hepatocellular ballooning and no residual lobular inflammation) with no worsening of fibrosis did not meet statistical significance in the ITT population (25 [8%] patients in the placebo group vs. 35 [11%] in the OCA 10 mg group [*p* = 0.18] or 36 [12%] in the OCA d 25 mg group [*p* = 0.13]). Pruritus occurred in 19% of the placebo group, 28% of patients treated with OCA 10 mg/die, and 51% of patients treated with OCA 25 mg/die.

### 3.1. OCA Worsens Lipoprotein and Lipid Profile in MASH Patients

Both the FLINT [112] and REGENERATE [113] studies have shown that OCA increases LDL-C while decreasing HDL-C in MASH patients compared to placebo. The new-onset dyslipidemia associated with OCA administration is highly common and occurs in almost all patients who are treated with OCA, as confirmed by the results of a randomized phase 2 study investigating the effects of OCA and atorvastatin on lipoproteins in MASH patients [121]. In this study, the CONTROL study, 84 patients with biopsy-confirmed MASH with no evidence of hepatic decompensation were randomly assigned to receive placebo or 5 mg, 10 mg, or 25 mg OCA once daily for 16 weeks. Concurrent once-daily atorvastatin (10 mg/die) was initiated at week 4 with subsequent titration. Confirming the negative impact of OCA on lipoprotein metabolism, at week 4, all OCA groups had an increase from baseline in mean LDL-C and mean LDL particle concentrations. Atorvastatin 10 mg decreased LDL-C and LDL particle concentration levels below baseline in all OCA groups by week 8, while higher doses did not provide additional clinical benefits. A further analysis of lipoprotein profiles of patients from the FLINT study [122] concluded that OCA therapy is associated with raised small VLDL and both large and small LDL particles, along with lowered HDL particles at 12 weeks, levels that were reverted to baseline values 24 weeks after drug interruption. While the mechanisms involved in worsening lipoprotein profile in response to OCA are not fully understood, treatment with atorvastatin effectively attenuates OCA-induced dyslipidemia. Further on, the detrimental increase in lipoprotein levels promoted by OCA improves/reverses after drug discontinuation, implying a specific FXR- or bile acid-dependent mechanism.

### 3.2. MASH Treatment and OCA

Despite the results of the REGENERATE study being negative in terms of NAS resolution and side effects, in 2019, Intercept Pharmaceuticals Ltd. applied to the FDA to receive an accelerate approval for OCA 25 mg/die in NASH patients. In June 2020, the FDA produced a complete response, based on the results from the REGENERATE study, claiming that the predicted benefits of OCA were not worth the potential risks in patients with fibrosis due to NASH and that long-term outcomes needed to be evaluated. Thus, an accelerated approval was not granted. Intercept applied for a second time in 2022 after a reanalysis of the primary endpoints of the REGENERATE trial by changing the method of analysis. However, an FDA Advisory Committee meeting on 29 May 2023 yielded negative votes, and a month later, a new Complete Response Letter was sent to Intercept Pharmaceuticals, calling again for a successful completion of the long-term outcomes phase of the REGENERATE study for resubmission of a New Drug Application for OCA in MASH. This time, the FDA raised major concerns related to OCA’s safety, concluding that despite showing some benefits in improving fibrosis in NASH patients, its use was associated with side effects such as pruritus, cholelithiasis, increased risk of drug-induced liver injury (DILI), dyslipidemia, and dysglycemia. A reanalysis of the OCA drug development program also detected an increase in the number of deaths: 17 deaths in patients treated with 25 mg OCA (1009 patients from various trials) compared to placebo (10 deaths from 1017 patients). The highest number of deaths, 14, occurred in the REGENERATE trial (https://www.fda.gov/media/168327/download, Accessed on 15 September 2025). Following this denial, on 24 June 2024, Intercept decided to discontinue all NASH-related operations, including a long-term patient outcome follow-up of the phase 3 REGENERATE study. The end-of-study results of the REGENERATE study on OCA treatment of pre-cirrhotic fibrosis due to MASH were presented at DDW in 2024 [123]. The results showed that patients receiving OCA 25 mg had reduced histological progression to cirrhosis, while in those taking OCA 10 mg, an improvement of ≥1 stage in fibrosis was observed. A reanalysis of the REGENERATE study was also published in 2023. The reanalysis concluded that OCA was superior to placebo in improving fibrosis using a more rigorous consensus panel analysis of liver biopsies taken at month 18 of the study [123]. The later re-analysis also showed that OCA treatment was associated with a dose-dependent amelioration ofliver biochemistries and liver stiffness compared to placebo, even in participants in whom histological fibrosis did not change at 18 months.

## 4. Other FXR Ligands in MASH

In addition to OCA, other FXR agonists, including cilofexor, vonafexor, and tropifexor, have been investigated in patients with MASH. Results from various phase 2 clinical trials are available. In these studies, MASH patients were treated with EDP-305 [14,124], tropifexor [125], vonaxefo [126], and cilofexor [127] (Table 6).

The efficacy of tropifexor in MASH has been examined in a phase 2 RCT, in which a 12–48-week intervention of this FXR agonist resulted in a dose-dependent reduction in ALT level in patients (−10.7 to −23.0 U/L under dosages ranging from 10 to 200 μg) [125]. Similarly to other FXR agonists, pruritus was the most common adverse effect observed in treated patients, and was more pronounced in patients receiving the higher doses of tropifexor. Cilofexor is a non-steroidal FXR agonist that has been investigated in patients with MASH, either alone or in combination with semaglutide and firsocostat [127,128,129]. In a double-blind [127], placebo-controlled phase 2 trial, 140 patients with noncirrhotic NASH, and steatosis diagnosed by magnetic resonance imaging–proton density fat fraction (MRI-PDFF) ≥ 8% and liver stiffness ≥ 2.5 kPa by magnetic resonance elastography (MRE) or historical liver biopsy, were randomized to receive cilofexor 100 mg (*n* = 56), 30 mg (*n* = 56), or placebo (*n* = 28) orally once daily for 24 weeks. Declines in MRI-PDFF of ≥30% were detected in 39% of patients receiving cilofexor 100 mg (*p* = 0.011 vs. placebo), 14% of those receiving cilofexor 30 mg, and 13% of those receiving placebo. Significant changes in Enhanced Liver Fibrosis (ELF) scores and liver stiffness were not observed. Moderate to severe pruritus was more common in patients receiving cilofexor 100 mg (14%) than in those receiving cilofexor 30 mg (4%) or placebo (4%). In another phase 2, open-label, proof-of-concept trial, 108 patients with NASH (F2-F3 on biopsy, or MRI-PDFF ≥ 10% and liver stiffness by transient elastography ≥ 7 kPa) were randomized to a 24-week treatment with semaglutide 2.4 mg once weekly as monotherapy or a combination of semaglutide once week with once-daily cilofexor (30 or 100 mg) and/or once-daily firsocostat 20 mg. Safety represented the primary endpoint, and all efficacy endpoints were exploratory. All treatments were well tolerated. Treatments achieved a similar weight loss (7–10%) compared with semaglutide monotherapy; however, a combination of treatments resulted in greater improvements in liver biochemistry, liver steatosis measured by MRI-PDFF (least-squares mean of absolute changes: −9.8 to −11.0% vs. −8.0%), and non-invasive tests of fibrosis. The authors concluded that the combination of cilofexor and firsocostat was safe and may offer additional benefits over treatment with semaglutide alone.

In a recent metanalysis [130] that included results from 29 published randomized controlled trials (*n* = 9324 patients) comparing pharmacological interventions in patients with biopsy-proven MASH, using as co-primary endpoints fibrosis improvement ≥ 1 stage without MASH worsening and MASH resolution without fibrosis worsening, it was found that all the drugs taken into consideration (pegozafermin, cilofexor + firsocostat, denifanstat, survodutide, obeticholic acid, tirzepatide, resmetirom, and semaglutide) allowed patients to achieve a significant regression of fibrosis without worsening MASH compared to placebo, with pegozafermin and cilofexor + firsocostat being the most effective interventions. Additionally, pegozafermin, survodutide, tirzepatide, efruxifermin, liraglutide, vitamin E + pioglitazone, resmetirom, semaglutide, pioglitazone, denifanstat, semaglutide, and lanifibranor showed better results in achieving MASH resolution without worsening fibrosis in comparison to placebo.

### MASH Treatment: The Current Landscape

Resmetirom has been the first drug approved for treatment of MASH in 2024 based on favorable results from the MAESTRO-NASH trial [131,132]. Resmetirom is a selective thyroid hormone receptor (THR)β agonist, and has received conditional approval in the USA (under the accelerated approval program), when administered together with diet and exercise, for the treatment of adult MASH patients without cirrhosis but with moderate (F2) to advanced (F3) liver fibrosis. In the EU, Resmetirom is under regulatory review for the treatment of MASH/NASH. In addition to resmetirom, various other agents are currently being investigated in phase 2 and 3 trials, including GLP-1 receptor agonists like semaglutide—a dual GLP-1 and glucagon receptor agonist—survodutide, tirzepatide, FGF21 analogs like efruxifermin and pegozafermin, and pan-PPAR agonists, such as lanifibranor (Table 7).

In June 2025, positive results from a phase 3 trial on semaglutide in MASH were reported. The results of the ESSENCE study [133] have provided a basis for conditional approval of semaglutide (marketed as Wegovy) for the treatment of adults with MASH and moderate to advanced liver fibrosis on 15 August 2025 (https://www.fda.gov/drugs/news-events-human-drugs/fda-approves-treatment-serious-liver-disease-known-mash#:~:text=The%20U.S.%20Food%20and%20Drug,and%20its%20prevalence%20is%20expanding, Accessed on 15 September 2025). The interim results of the ESSENCE study demonstrated that 63% of participants receiving semaglutide experienced MASH resolution and no worsening of fibrosis compared to 34% of the participants receiving placebo. Furthermore, 37% of participants on semaglutide saw improvements in fibrosis and no worsening of MASH, compared to 22% of the participants receiving placebo [133].

**Table 7:** Drugs currently under investigation for NASH/MASH treatment.

Compound	Molecular Target	References
Lanifibranor	Pan-PPAR	[134,135]
Efruxifermin	FGF21 analog	[136]
Pegozafermin	FGF21 analog	[137]
Survodutide	GLP-1R/GCGR dual agonist	[138]

Results for some of these agents will be available in the next 2–3 years.

## 5. Conclusions

As of September 2025, the market authorization of OCA is restricted to therapy for non-cirrhotic adult PBC patients who either have an inadequate response to or cannot tolerate UDCA. However, in the EU, its marketing authorization has been revoked due to the lack of efficacy and safety concerns generated by the results of the COBALT trial. Concerns over OCA safety, including hepatic decompensation and death in cirrhotic and non-cirrhotic PBC patients are impacting the future perspectives of using OCA in PBC patients. Further on, the recent approval of seladelpar and elafibranor poses a significant challenge to OCA’s continued clinical utility as a second-line treatment in PBC. Currently, OCA remains available in number of countries (see Table 8).

The clinical development of OCA in MASH has been discontinued because of safety concerns and suboptimal efficacy. While OCA consistently demonstrated an antifibrotic effect in clinical trials, its impacts on other histological components of the disease, including hepatic steatosis, ballooning, and lobular inflammation, were limited. In the two main clinical trials, the FLINT and the REGENERATE trials, involving patients with biopsy-proven MASH, OCA failed to achieve statistically significant improvement in overall steatohepatitis or resolution of the NAFLD Activity Score (NAS). This is the reason why, following current regulatory guidelines, its approval for MASH indications has been denied. These limited therapeutic benefits are offset by a concerning safety profile. OCA administration has been associated with a spectrum of adverse effects, some of which are serious, including hepatic decompensation, liver transplantation, and mortality. These risks are particularly pronounced in patients with pre-existing cirrhosis, leading to a formal contraindication for its use in cirrhotic individuals, irrespective of etiology. Notably, severe cases of DILI and hepatic decompensation have also been reported in non-cirrhotic patients, further narrowing its therapeutic window. In the REGENERATE program, the use of OCA is associated with an excess of mortality in comparison to placebo. While side effects and lack of efficacy have led to the discontinuation of the MASH development program, the antifibrotic activity of OCA has been confirmed in various NASH/MASH trials, suggesting a potential role for OCA as part of combination regimens, although the recent approval of resmetirom and semaglutide for treatment of MASH is likely to impact the future development of FXR agonists. However, given that fibrosis severity is the strongest predictor of liver-related outcomes in MASH patients, that many investigational therapies have failed to meet fibrosis-related endpoints, and that all FXR agonists, tropifexor, and cilefexor ameliorate liver fibrosis, a likely scenario could be the development of combinatorial therapies to treat fibrosis and steatosis in MASH patients. Nonetheless, no ongoing clinical trials are currently exploring this approach, limiting the near-term prospects for further development of FXR ligands for this indication.

## Figures and Tables

**Table 1:** Summary of studies showing efficacy of OCA (6ethyl-CDCA or INT-747) in rodent models.

Animal Model	Dose Tested	Main Findings	Refs.
Liver Fibrosis			
Bile duct ligation	3 mg/kg	Attenuates liver fibrosis. Worsens cholestasis.	[26]
Porcine serum	1–10 mg/kg	Attenuates liver fibrosis.	[26]
Carbon tetrachloride (CCL_4)_	3 mg/kg	Attenuates liver fibrosis.	[27]
Thioacetamide (TAA)		Attenuates liver fibrosis.	[36]
CCL4, bile duct ligation and DIO		Prophylactic but not therapeutic administration of obeticholic acid (OCA) prevents hepatic stellate cell (HSC) activation and fibrogenesis.	[37]
LDLr^−/−^ mice feed HFD		Reduces fibrosis.	[38]
TAA		Reduces fibrosis.	[36]
Ob/ob mice fed with the Control diet or amylin liver NASH (AMLN) diet		OCA, Ferrostatin-1, and their combination improve steatosis and fibrosis.	[39]
BDL	40 mg/kg	OCA worsens fibrosis.	[40]
**Cholestasis**			
Estrogen-induced cholestasis	3 mg/kg	Attenuates non-obstructive cholestasis.	[41]
Bile duct ligation (BDL)	3 mg/kg	Worsens obstructive cholestasis.	[26]
**Portal hypertension**			
CCL_4_	10 mg/kg	Reduces portal pressure.	[42]
BDL and TAA	30 mg/kg	Reduces portal pressure.	[43]
BDL	5 mg/kg	OA treatment significantly increased DDAH-1 expression, reduced hepatic tissue ADMA, and increased liver NO generation.	[44]
**Liver immunity**			
Concanavalin A	10 mg/kg	Attenuates liver injury by an NKT mechanism.	[45]
Sepsis-induced liver dysfunction	10 mg/kg	OCA attenuates liver injury in a model of intestinal perforation-induced sepsis.	[46]
**Non-alcoholic steatohepatitis (NASH)—Metabolic Associated Liver Disease (MASLD)**			
Genetic models			
Zucker rats	3 mg/kg/d	Ameliorates insulin sensitivity.	[47]
Apo E^−/−^	3–10 mg/kg/d	Attenuates NAS. Reduces HDL.	[28]
Diet-induced NASH	OCA and miR-21	miR-21 abrogation, together with FXR activation by OCA, significantly improves whole-body metabolic parameters in NASH, o effect cholesterol.	[48]
Diet-induced NASH	OCA and GLP-1 agonist	GLP-1R agonist and OCA exert synergistic effects in mouse models of metabolic disease and NASH.	[49]
Diet-induced NASH	15 mg/kg	OCA reduces inflammation and fibrosis but not total NAS and increases LDL-c.	[50]
Diet-induced obesity in wild-type and ob/ob mice	OCA, liraglutide and elafibranor 30 mg/kg	Liraglutide and elafibranor, but not OCA, reduced body weight in both models. Liraglutide improved steatosis scores in DIO-NASH mice only. Elafibranor and OCA reduced histopathological scores of hepatic steatosis and inflammation in both models, but only elafibranor reduced fibrosis severity.	[51]
Diet-induced NASH in wild-type and ob/ob mice	OCA and elafibranor 30 mg/kg	OCA and elafibranor synergize in ameliorating liver BAs and fibrosis score in diet model and ob/ob mice.	[52]
**Kidney in jury**			
Diet-induced kidney injury	OCA 10 mg/kg	Ameliorates triglyceride accumulation by modulating fatty acid synthesis and oxidation, and improves proteinuria, accumulation of extracellular matrix proteins, and profibrotic markers.	[53]
**Adipose tissue**			
Diet-induced metabolic syndrome and NASH	10 mg/kg	OCA improved adipose tissue morphology, glucose tolerance, and steatosis in a milder metabolic phenotype but failed to improve these factors in morbidly obese diabetic mice.	[54]
**Intestinal inflammation**			
Dextran sodium sulfate (DSS)- and Trinitrobenzene sulfonic acid (TNBS)-induced colitis. Wild-type and Fxr./-mice		OCA abrogates intestinal inflammation via inhibition of NFkb.	[29]
DSS and TNBS		OCA attenuates inflammation by modulating CD14 monocytes.	[55]
BDL rats	5 mg/day	OCA activates the intestinal FXR signaling pathway and improves the composition and structure of the intestinal microbiota and intestinal barrier in BDL rats.	[56]
Diet-induced NASH		OCA protects against intestinal barrier disruption and prevents the development of NASH.	[57]
CCl_4_-induced cirrhosis with ascites.	0.5 mg/kg	In ascitic cirrhotic rats, OCA reduces gut bacterial translocation.	[58]
**Intestinal microbiota**			
Short bowel syndrome		CA supplementation could effectively ameliorate the intestinal barrier disruption and inhibit overexpression of pro-inflammatory factors.	[59]
Diet-induced steatosis		OCA ameliorates intestinal permeability.	[60]
Healthy volunteers and intact mice	5, 10 and 25 mg	OCA remodels intestinal microbiota and increases the proportion of *Firmicutes* in mice.	[61]
Diet-induced obesity and *C. difficile* infection		OCA attenuates *C. difficile* infection in obese mice.	[62]
**Pulmonary fibrosis**			
Bleomycin	3–10 mg/kg	Improves pulmonary fibrosis and ventilatory function.	[63]
**Psychological stress-related injury**			
Diet-induced steatosis		OCA ameliorates anxiety behavior related to steatosis and diet-induced intestinal dysbiosis.	[64]
**Colon cancer**			
		OCA combined with β-catenin inhibitors attenuates colon cancer progression.	[65]
		OCA promoted SOCS3 transcription by enhancing the binding of FXR to the FXRE/IR9 of the SOCS3 promoter.	[66]

**Table 3:** Currently available treatments for PBC.

	Drug	Molecular Target	Dosage	Reference
**First-line**	Ursodeoxycholic acid (UDCA)	GPBAR1 agonist, FXR partial antagonist	13–15 mg/kg/die, PO	[70,72,75]
**Second-line**	Seladelpar (^®^Livdelzi)	PPARδ agonist	10 mg/die, PO	[107,108]
	Elafibranor (^®^Iqirvo)	PPARα/δ agonist	80 mg/die, PO	[106]
	Bezafibrate	Pan-PPAR agonist	400 mg/die, PO	[109,110]
	Fenofibrate	PPARα agonist	100–200 mg/die, PO	[111]
	Saroglitazar	PPARα/γ agonist	1–2 mg/die, PO	
	Obeticholic acid (OCA) (^®^Ocaliva)	FXR agonist	5 mg/die up to 10 mg/die in IR after 6 months, PO	[67,68,84,85,86,89,112,113]
	Budesonide	Glucocorticoid receptor agonists	Under evaluation Placebo-controlled RCT: 9 mg/die	[114]
	Prednis(ol)one		ND	[115]

**Table 4:** Metabolic effects of OCA in a phase 2 exploratory trial in diabetic patients. Data shown are the results at day 43 after randomization [101].

Analyte	Placebo (n. 23)	OCA 25 mg/d (n. 20)	OCA 50 mg/d (n. 21)
FGF19 (ng/L)	91 ± 11	177 ± 23 *	255 ± 42 *
Alk. Phos.	77 ± 21	86 ± 37 *	103 ± 36 *
LDL	107 ± 34	120 ± 31 *	129 ± 35 *
HDL	40 ± 10	35 ± 6	37 ± 7 *
TG	178 ± 90	170 ± 81	121 ± 50 *

* *p* ≤ 0.05.

**Table 5:** Clinical trials on OCA in MASH patients.

Study ID (Clinical Phase)	Groups	Fibrosis Improvement ≥ 1 Stage No NASH Worsening	NASH Resolution * (Not Significant = 0.08)	NAS Resolution with No Fibrosis Worsening	Pruritus	Ref.
**NCT01265498** **FLINT study** **(Phase 2)**	OCA 25 mg/d Placebo	35% vs. 19% (0.004)	22% vs. 13% Not significant (0.08)		22.7 vs. 6.3%	[112]
**NCT02548351** **REGENERATE** **(Phase 3)**	OCA 10 mg/d (*n* = 312) OCA 25 mg/d (*n* = 308) Placebo (*n* = 311)	ITT = 18% vs. 23% * vs. 12.0% (* 0.0002 vs. placebo)		12% vs. 11% vs. 8% (not significant)	35.6% vs. 56.8% vs. 25.4%	[113]

* NASH resolution: ballooning NAS = 0; inflammation NAS = 0–1.

**Table 6:** FXR agonists currently being developed for treatment of MASH.

	EDP-305 [124]			Cilofexor [127]		Tropifexor [125]			Vonafexor [126]		
**Clinical trials** **Phase**	NCT03421431 Phase 2			NCT03449446 Phase 2		NCT02855164 Phase 2			NCT03812029 Phase 2		
**Doses [mg]** **(P: Placebo)**	P	1	2.5	P	30	P	0.14	0.2	P	100	200
**Percent of** **Fibrosis** **improvement**	−0.18	0.6	0.28	11	12 ^ns^	35	38	68	0	−0.06 *	−0.03 *
**Percent of NASH** **improvement**	2.4	3.3 ^ns^	7.1 ***	-	N/A	6.19	19.07 ***	39.41 ***	2.3	6.3 **	5.4 *
**Percent of** **pruritus**	4.2	9.1	50.9	15.3	20	22	52	69	6.3	9.7	18.2

* *p* ≤ 0.05, ** *p* ≤ 0.01, *** *p* ≤ 0.001.

**Table 8:** Current status of OCA commercialization in selected countries around the world. Commercialization is currently limited by country-specific regulations and limitations.

Countries	Market Availability of OCA Under Various Brands
USA and Canada	YES
South America	NO
EU	NO
UK	YES
Russia	YES
Australia	YES
Africa South Africa, Kenya Egypt, Nigeria, Congo	Not widely available YES NO
ASIA Japan, China, India, Philippines, Singapore, Indonesia and Republic of Korea	YES
ASIA Taiwan, Turkey, Pakistan, Thailand	NO

## Data Availability

Not applicable.
